# Supplementary material for: Consistent diel activity patterns of forest mammals among tropical regions
Source: Nat Commun. 2022 Nov 19;13:7102. doi: 10.1038/s41467-022-34825-1 (PMC9675769; doi:10.1038/s41467-022-34825-1)
Supplement: Supplementary file 1 — Supplementary Information [file 41467_2022_34825_MOESM1_ESM.docx]

**Supplementary Information**

Table S1. Additional information about the protected areas included in the study.

Table S2. Candidate multinomial models ranked according to Akaike information criterion (AIC) for each biogeographic region.

Table S3. Summary from the multinomial logit model on each biogeographic area.

Figure S1. Multinomial model coefficient estimates by each region.

Figure S2. a) Distribution of body mass for the three different biogeographic regions. b) Number of species in each trophic guild and each biogeographic region.

Figure S3. Examples of Kernel density activity of species by tropical region and trophic group.

Figure S4. Extended predicted probability of being diurnal (a), crepuscular (b) and nocturnal (b) for a sequence of body mass values by trophic guild and continent.

Figure S5 – Figure S12. Kernel density plots of the activity of all species groups and overlap estimates.

**Table S1. Additional information about protected areas included in the study.**

| **Protected area** | **Code** | **Area (ha)*** | **Country** | **Tropical region** | **Number of cameras** | **Number of Families**** | **Number of Species**** | **Presence of top-predators** |
| --- | --- | --- | --- | --- | --- | --- | --- | --- |
| Barro Colorado Nature Monument - Soberania National Park | BCI | 4,890 | Panama | Neotropical | 60 | 15 | 24 | No |
| Bukit Barisan | BBS | 295,000 | Indonesia | Indo-Malayan | 60 | 18 | 32 | Yes |
| Bwindi Impenetrable Forest | BIF | 32,700 | Uganda | Afrotropical | 60 | 13 | 22 | No |
| Caxiuanã National Forest | CAX | 317,945 | Brazil | Neotropical | 60 | 12 | 23 | Yes |
| Central Suriname Nature Reserve | CSN | 1,600,000 | Suriname | Neotropical | 65 | 13 | 30 | Yes |
| Cocha Cashu - Manu National Park | COU | 1,716,294 | Peru | Neotropical | 60 | 15 | 32 | Yes |
| Korup National Park | KRP | 125,900 | Cameroon | Afrotropical | 60 | 11 | 17 | No |
| Manaus | MAS | 1,198,944 | Brazil | Neotropical | 89 | 11 | 22 | Yes |
| Nam Kading | NAK | 169000 | Laos | Indo-Malayan | 60 | 16 | 25 | No |
| Nouabalé Ndoki | NNN | 423,870 | Republic of Congo | Afrotropical | 64 | 16 | 30 | Yes |
| Pasoh Forest Reserve | PSH | 13,610 | Malaysia | Indo-Malayan | 60 | 18 | 34 | Yes |
| Udzungwa | UDZ | 208,869 | Tanzania | Afrotropical | 61 | 15 | 24 | Yes |
| Virunga Massif | VIR | 16,000 | Rwanda-Uganda | Afrotropical | 60 | 7 | 10 | No |
| Volcán Barva (Brulio Carrillo National Park) | VB | 49,921 | Costa Rica | Neotropical | 60 | 13 | 22 | Yes |
| Yanachaga Chimillen National Park | YAN | 122,000 | Peru | Neotropical | 63 | 14 | 25 | Yes |
| Yasuni National Park | YAS | 1,030,070 | Ecuador | Neotropical | 60 | 13 | 28 | Yes |

*Areas were extracted from the World Database on Protected Areas (WDPA). Pasoh Forest Reserve, and Manaus were extracted from Beaudrot et al. 2017 67.

**Number of mammals families and species detected by camera traps and included in this study. Species with a body mass >=75 g, ground dwelling and scansorial species

**Table S2. Candidate multinomial models ranked according to Akaike information criterion (AIC) for each biogeographic region.** LR= Likelihood ratio**,** AIC=Akaike Information Criterion, N= sample size (number of independent events), and ΔAIC= delta AIC.

| **Region** | **Model** | **N** | **LR** | **AIC** | **ΔAIC** |
| --- | --- | --- | --- | --- | --- |
| Afrotropics | trophic_guild * b_mass + 1\|Protected.area | 60704 | 51357.51 | 82054.81 | 0.00 |
| Afrotropics | trophic_guild + b_mass+ 1\|Protected.area | 60704 | 47432.70 | 85967.62 | 3912.80 |
| Afrotropics | trophic_guild+ 1\|Protected.area | 60704 | 45910.99 | 87485.33 | 5430.52 |
| Afrotropics | b_mass+ 1\|Protected.area | 60704 | 33107.59 | 100280.73 | 18225.92 |
| Afrotropics | 1+ 1\|Protected.area | 60704 | 29922.11 | 103462.21 | 21407.40 |
| Indo-Malayan | trophic_guild * b_mass+ 1\|Protected.area | 8602 | 6102.54 | 12829.98 | 0.00 |
| Indo-Malayan | trophic_guild + b_mass+ 1\|Protected.area | 8602 | 5677.21 | 13243.32 | 413.33 |
| Indo-Malayan | trophic_guild+ 1\|Protected.area | 8602 | 5639.64 | 13276.88 | 446.90 |
| Indo-Malayan | b_mass+ 1\|Protected.area | 8602 | 5318.54 | 13589.99 | 760.01 |
| Indo-Malayan | 1+ 1\|Protected.area | 8602 | 5299.85 | 13604.68 | 774.69 |
| Neotropics | trophic_guild * b_mass+ 1\|Protected.area | 57076 | 37722.82 | 87717.97 | 0.00 |
| Neotropics | trophic_guild + b_mass+ 1\|Protected.area | 57076 | 31624.34 | 93804.45 | 6086.48 |
| Neotropics | trophic_guild+ 1\|Protected.area | 57076 | 30988.40 | 94436.39 | 6718.42 |
| Neotropics | b_mass+ 1\|Protected.area | 57076 | 24355.06 | 101061.73 | 13343.76 |
| Neotropics | 1+ 1\|Protected.area | 57076 | 24044.83 | 101367.96 | 13649.99 |

**Table S3.** S**ummary from the multinomial logit model on each biogeographic area.** Values represent the mean coefficient from the model and the significance, the p-value from the Wald-test, standard error are within parenthesis.

| 1. **Neotropics** |  | | |  | | | |
| --- | --- | --- | --- | --- | --- | --- | --- |
|  | night/day | | | twilight/day | | | |
| (Intercept) | 1 | . | 613*** | 0 | . | | 095 |
|  | (0 | . | 336) | (0 | . | | 337) |
| logmassk | −0 | . | 503*** | −0 | . | | 539*** |
|  | (0 | . | 053) | (0 | . | | 092) |
| trophic_guild: Herbivores/Carnivores | −2 | . | 888*** | −2 | . | | 159*** |
|  | (0 | . | 157) | (0 | . | | 257) |
| trophic_guild: Insectivores/Carnivores | 4 | . | 333*** | 0 | . | | 600 |
|  | (0 | . | 231) | (0 | . | | 410) |
| trophic_guild: Omnivores/Carnivores | 1 | . | 562*** | 0 | . | | 210 |
|  | (0 | . | 190) | (0 | . | | 289) |
| logmassk x trophic_guild: Herbivores/Carnivores | 0 | . | 846*** | 0 | . | | 587*** |
|  | (0 | . | 053) | (0 | . | | 093) |
| logmassk x trophic_guild: Insectivores/Carnivores | −1 | . | 240*** | −0 | . | | 363* |
|  | (0 | . | 087) | (0 | . | | 170) |
| logmassk x trophic_guild: Omnivores/Carnivores | −3 | . | 651*** | −1 | . | | 911*** |
|  | (0 | . | 118) | (0 | . | | 156) |
| night/day x VCov(~1,~1) | 0 | . | 704 | 0 | . | | 171 |
|  | (0 | . | 212) | (0 | . | | 096) |
| twilight/day x VCov(~1,~1) | 0 | . | 171 | 0 | . | | 383 |
|  | (0 | . | 096) | (0 | . | | 052) |
| Groups by Site.Code | 8 |  |  |  |  | |  |
| Deviance | 84205 | . | 7 |  |  | |  |
| N | 57076 |  |  |  |  | |  |
| Significance: *** = p < 0.001; ** = p < 0.01; * = p < 0.05 | | | | | | | |
| 1. **Afrotropics** |  | | |  | | | |
|  | night/day | | | twilight/day | | | |
| (Intercept) | 2 | . | 341*** | 0 | . | | 176 |
|  | (0 | . | 212) | (0 | . | | 235) |
| logmassk | −0 | . | 845*** | −0 | . | | 521*** |
|  | (0 | . | 053) | (0 | . | | 070) |
| trophic_guild: Herbivores/Carnivores | −4 | . | 406*** | −3 | . | | 585*** |
|  | (0 | . | 120) | (0 | . | | 157) |
| trophic_guild: Insectivores/Carnivores | −1 | . | 347*** | −1 | . | | 212*** |
|  | (0 | . | 130) | (0 | . | | 168) |
| trophic_guild: Omnivores/Carnivores | 1 | . | 090*** | −0 | . | | 406* |
|  | (0 | . | 133) | (0 | . | | 175) |
| logmassk x trophic_guild: Herbivores/Carnivores | 1 | . | 216*** | 0 | . | | 889*** |
|  | (0 | . | 053) | (0 | . | | 071) |
| logmassk x trophic_guild: Insectivores/Carnivores | 3 | . | 744*** | 1 | . | | 823*** |
|  | (0 | . | 133) | (0 | . | | 160) |
| logmassk x trophic_guild: Omnivores/Carnivores | −1 | . | 300*** | −0 | . | | 271** |
|  | (0 | . | 082) | (0 | . | | 102) |
| night/day x VCov(~1,~1) | 0 | . | 158 | 0 | . | | 007 |
|  | (0 | . | 003) | (0 | . | | 002) |
| twilight/day x VCov(~1,~1) | 0 | . | 007 | 0 | . | | 165 |
|  | (0 | . | 002) | (0 | . | | 003) |
| Groups by Site.Code | 5 |  |  |  |  | |  |
| Deviance | 81612 | . | 5 |  |  | |  |
| N | 60704 |  |  |  |  | |  |
| Significance: *** = p < 0.001; ** = p < 0.01; * = p < 0.05 | | | | | | | |
| 1. **Indo-Malayan tropics** |  | | |  | | | |
|  | night/day | | | twilight/day | | | |
| (Intercept) | 0 | . | 585 | −4 | . | 295*** | |
|  | (0 | . | 661) | (1 | . | 252) | |
| logmassk | −0 | . | 375** | 0 | . | 601* | |
|  | (0 | . | 135) | (0 | . | 271) | |
| trophic_guild: Herbivores/Carnivores | −1 | . | 877*** | 1 | . | 781 | |
|  | (0 | . | 332) | (1 | . | 140) | |
| trophic_guild: Insectivores/Carnivores | 0 | . | 418 | 3 | . | 945** | |
|  | (0 | . | 625) | (1 | . | 267) | |
| trophic_guild: Omnivores/Carnivores | 0 | . | 976** | 2 | . | 641* | |
|  | (0 | . | 355) | (1 | . | 185) | |
| logmassk x trophic_guild: Herbivores/Carnivores | 0 | . | 551*** | −0 | . | 465 | |
|  | (0 | . | 136) | (0 | . | 273) | |
| logmassk x trophic_guild: Insectivores/Carnivores | 3 | . | 540*** | 0 | . | 824 | |
|  | (0 | . | 454) | (0 | . | 448) | |
| logmassk x trophic_guild: Omnivores/Carnivores | −0 | . | 570*** | −0 | . | 556 | |
|  | (0 | . | 163) | (0 | . | 306) | |
| night/day x VCov(~1,~1) | 0 | . | 987 | 0 | . | 474 | |
|  | (1 | . | 372) | (1 | . | 115) | |
| twilight/day x VCov(~1,~1) | 0 | . | 474 | 0 | . | 831 | |
|  | (1 | . | 115) | (1 | . | 032) | |
| Groups by Site.Code | 3 |  |  |  |  |  | |
| Deviance | 12459 | . | 6 |  |  |  | |
| N | 8602 |  |  |  |  |  | |
| Significance: *** = p < 0.001; ** = p < 0.01; * = p < 0.05 | | | | | | | |

**
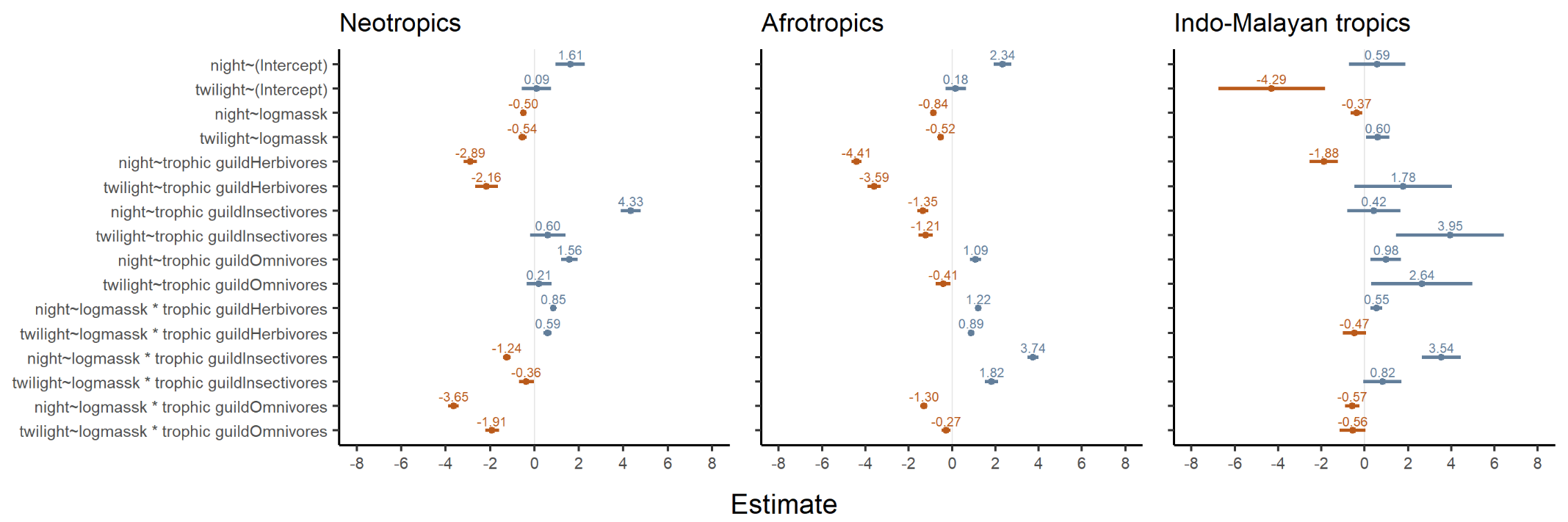
**

**Figure S1.** **Multinomial model coefficient estimates by each region.** The intercept (vertical line at 0) is represented with Carnivores as the reference group. Bars represent the 95% confidence intervals. Significance was considered when the 95 % CI did not overlap zero (horizontal lines). Neotropics n=57076, Afrotropics n=60704, Indo-Malayan tropics n=8602. n represent the number of independent events employed to fit the multinomial models at the regional level.


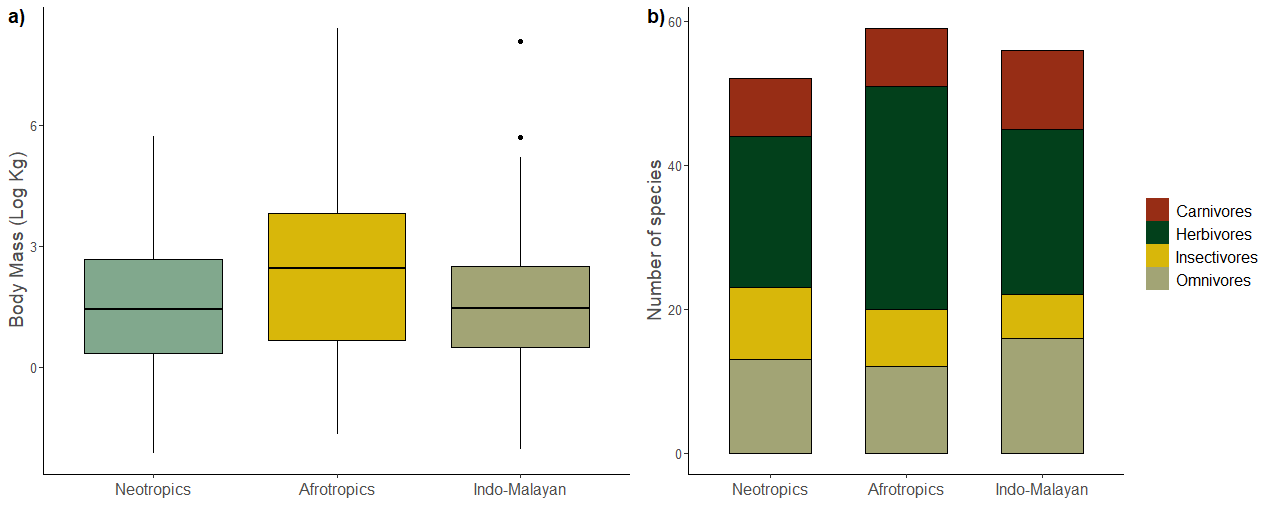


**Figure S2.** a) Distribution of body mass values (log scale kg) of ground-dwelling mammals for the three different biogeographic regions, boxplot centres represent the median, hinges represent the 25th and 75th percentiles, whisker extend from the hinges to the largest and smallest value no further than 1.5 times the interquartile from the hinge. b) Number of species in each trophic guild and each biogeographic region. Neotropics n=52, Afrotropics n= 59, Indo-Malayan tropics n= 56 (n= number of species).

**
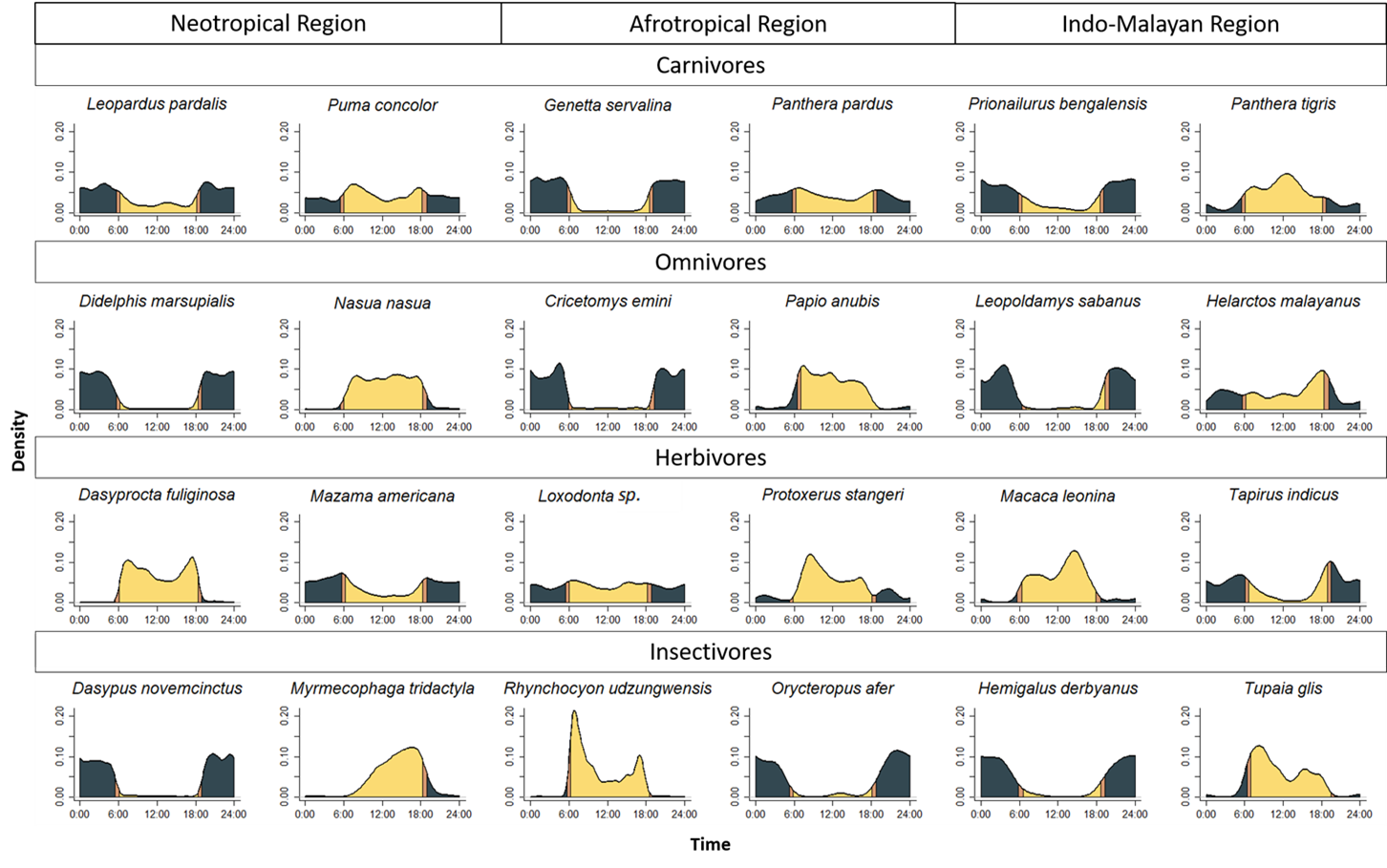
**

**Figure S3.** **Examples of Kernel density activity of species by tropical region and trophic group.** Yellow, dark blue, and orange represents day, night, and twilight, respectively.


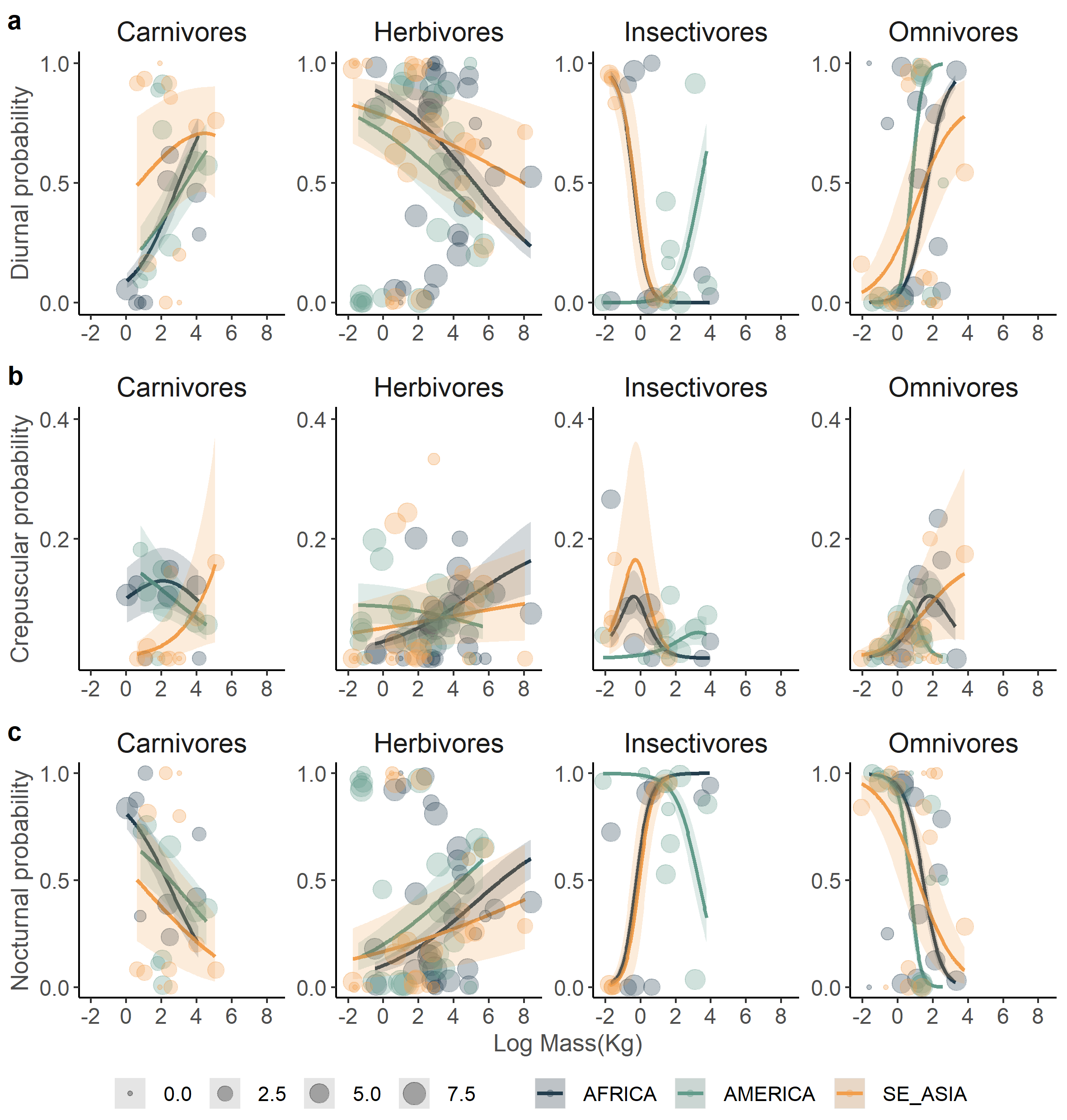


**Figure S4.** **Extended predicted probability of being diurnal (a), crepuscular (b) and nocturnal (b) for a sequence of body mass values by trophic guild and continent.** Lines represent continuous predictions along a range of body mass values extracted from the fitted multinomial logit models. Lighter regions correspond to the upper and lower 95% confidence intervals. Circles represent raw proportions of diurnal, crepuscular, and nocturnal activity for species included in this study are based on the number of independent events (log-scaled), with the smallest circle corresponding to 1 and the biggest to 1055 events. Total number of independent events: 126 382. n_carnivores_Neotropics_= 2182, n_carnivores_Afrotropics_= 1474, n_carnivores_Indo-Malayan_tropics_ = 152, n_omnivores_Neotropics_ = 4656, n_omnivores_Afrotropics_= 4656, n_omnivores_Indo-Malayantropics_= 435, n_herbivores_Neotropics_= 45839, n_herbivores_Afrotropics_= 47458, n_herbivores _Indo-Malayan_tropics_= 7803, n_insectivores_Neotropics_= 4399, n_insectivores_Afrotropics_ = 3886, n_insectivores_Indo-Malayan_tropics_ = 212.


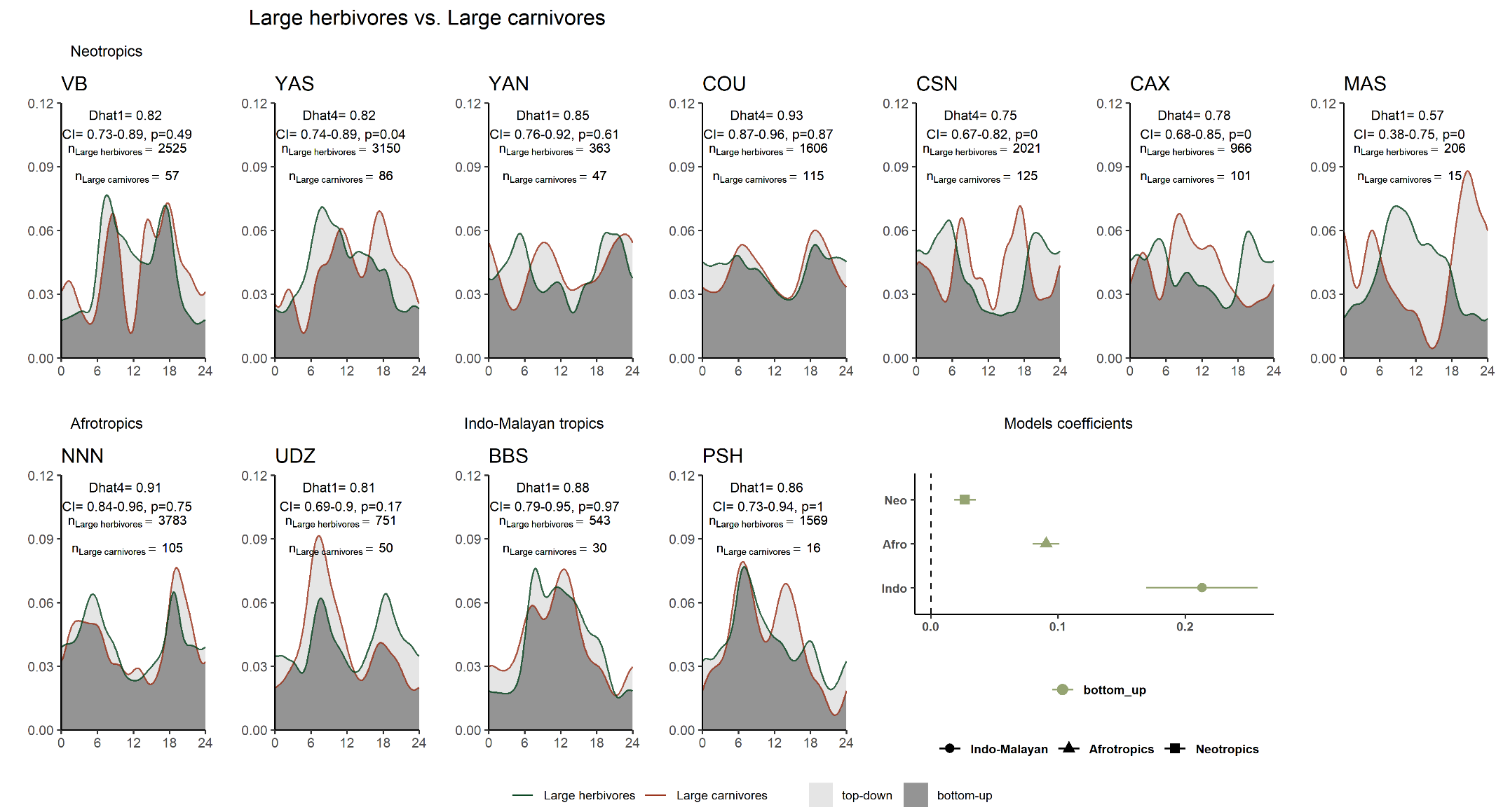


**Figure S5.** Fitted kernel density distribution and coefficient of overlap (Dhat) between the activity of large carnivores vs. large herbivores at the protected area level. Lines: Fitted kernel density distribution of each group, Dhat1: coefficient of overlap type 1 if the sample size was lower than 75, and Dhat4: type 1 if the sample size was higher than 75. CI: 95% confidence intervals, p-value: probability that the fitted distributions of the activity among these groups came from the same distribution. “n” represents the number of independent events in each trophic guild and size. Bottom right panel represents the results at the regional level employing GLMM, Figure 4 (Manuscript).


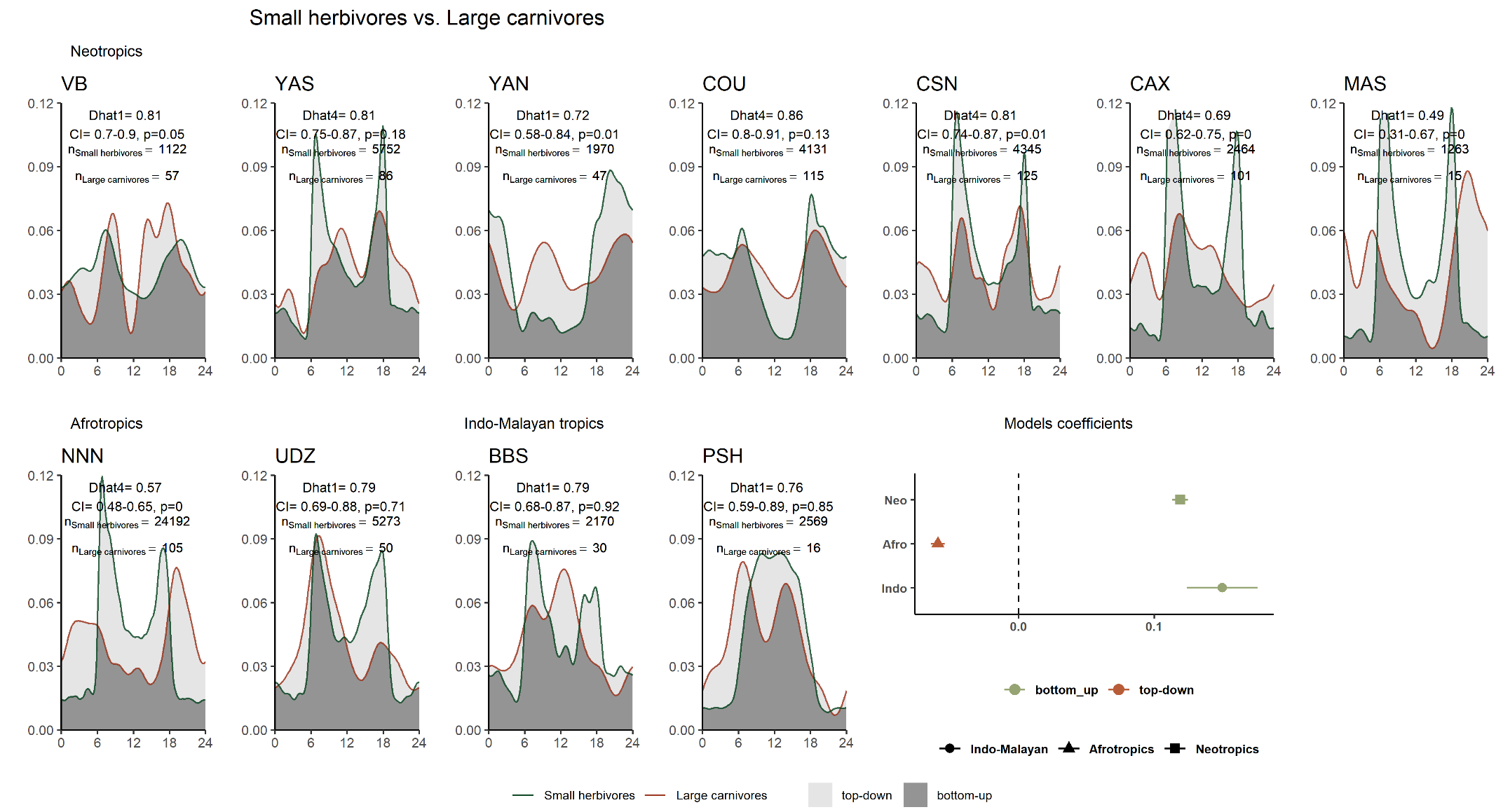


**Figure S6.** Fitted kernel density distribution and coefficient of overlap (Dhat) between the activity of large carnivores vs. small herbivores at the protected area level. Lines: Fitted kernel density distribution, Dhat1: coefficient of overlap type 1 if the sample size was lower than 75, and Dhat4: type 1 if the sample size was higher than 75, CI: 95% confidence intervals, p-value: probability that the fitted distributions of the activity among these groups came from the same distribution. “n” represents the number of independent events in each trophic guild and size. Bottom right panel represents the results at the regional level employing GLMM, Figure 4 (Manuscript).

**
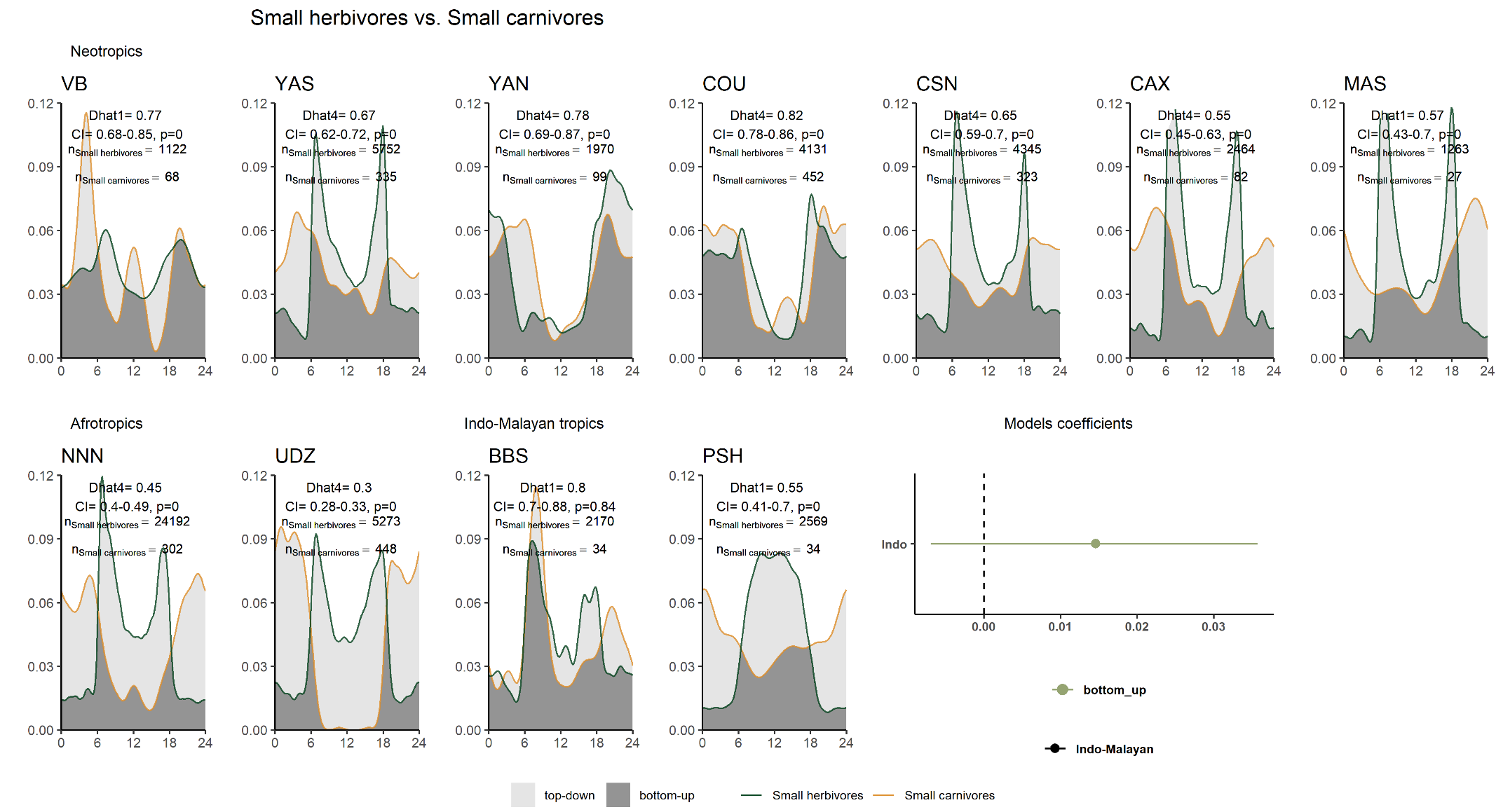
Figure S7.** Fitted kernel density distribution and coefficient of overlap (Dhat) between the activity of small carnivores vs. small herbivores at the protected area level. Lines: Fitted kernel density distribution, Dhat1: coefficient of overlap type 1 if the sample size was lower than 75, and Dhat4: type 1 if the sample size was higher than 75, CI: 95% confidence intervals, p-value: probability that the fitted distributions of the activity among these groups came from the same distribution. “n” represents the number of independent events in each trophic guild and size. Bottom right panel represents the results at the regional level Figure 4.

**
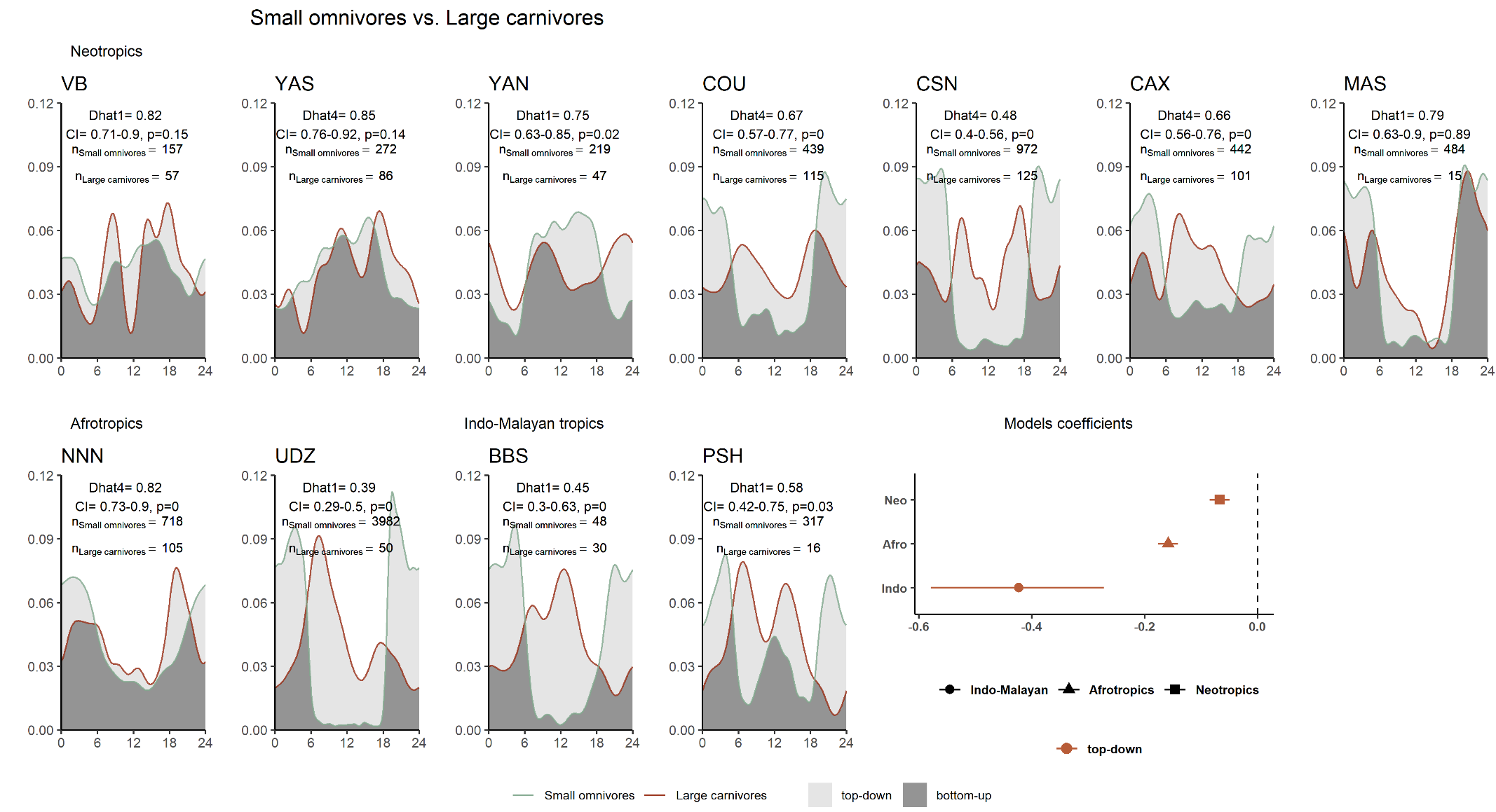
**

**Figure S8.** Fitted kernel density distribution and coefficient of overlap (Dhat) between the activity of large carnivores vs. small omnivores at the protected area level. Lines: Fitted kernel density distribution, Dhat1: coefficient of overlap type 1 if the sample size was lower than 75, and Dhat4: type 1 if the sample size was higher than 75, CI: 95% confidence intervals, p-value: probability that the fitted distributions of the activity among these groups came from the same distribution. “n” represents the number of independent events in each trophic guild and size. Bottom right panel represents the results at the regional level Figure 4.


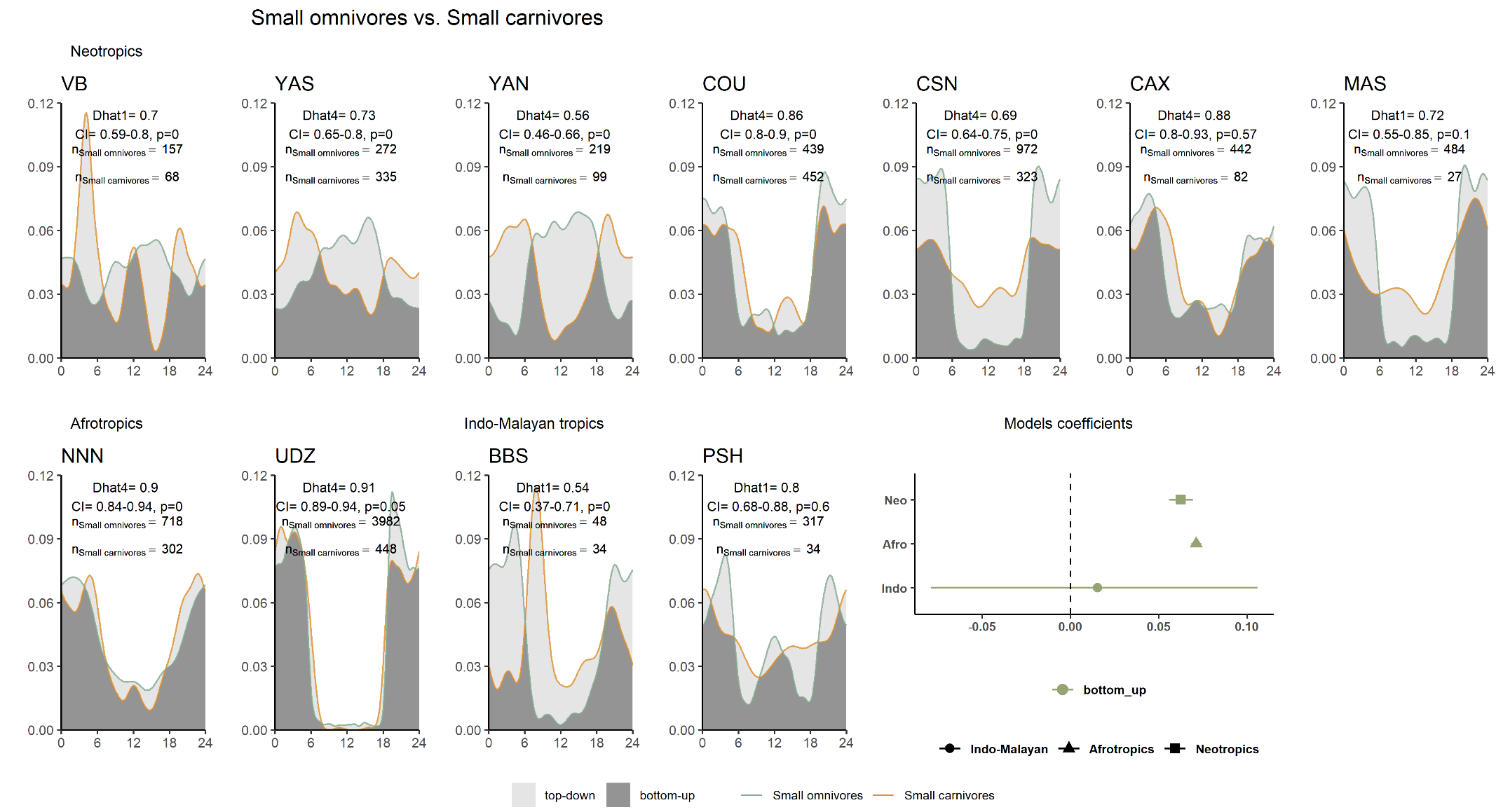


**Figure S9.** Fitted kernel density distribution and coefficient of overlap (Dhat) between the activity of small omnivores vs. small carnivores at the protected area level. Lines: Fitted kernel density distribution, Dhat1: coefficient of overlap type 1 if the sample size was lower than 75, and Dhat4: type 1 if the sample size was higher than 75, CI: 95% confidence intervals, p-value: probability that the fitted distributions of the activity among these groups came from the same distribution. “n” represents the number of independent events in each trophic guild and size. Bottom right panel represents the results at the regional level Figure 4.

**
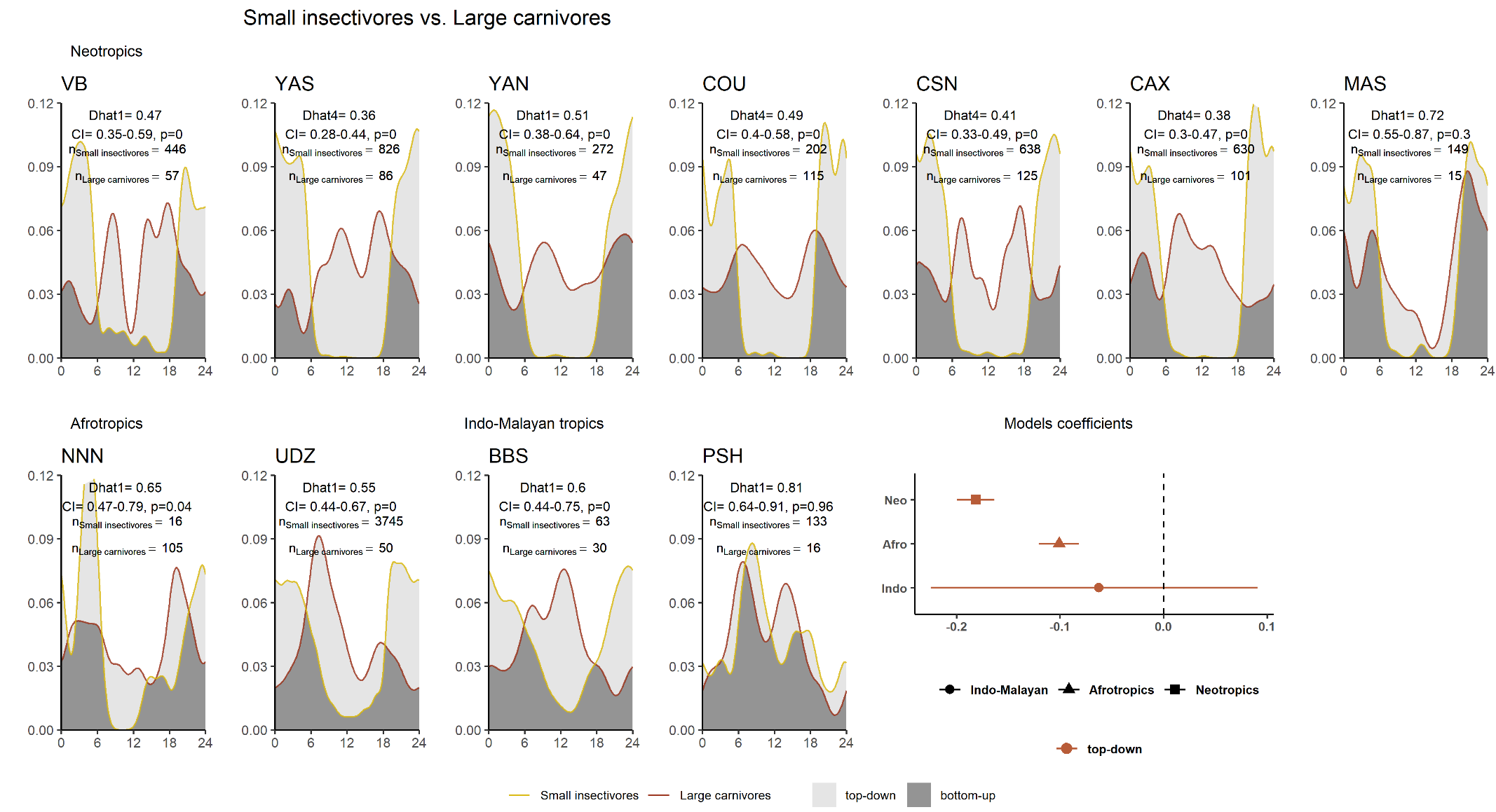
 Figure S10.** Fitted kernel density distribution and coefficient of overlap (Dhat) between the activity of large carnivores vs. small insectivores at the protected area level. Lines: Fitted kernel density distribution, Dhat1: coefficient of overlap type 1 if the sample size was lower than 75, and Dhat4: type 1 if the sample size was higher than 75, CI: 95% confidence intervals, p-value: probability that the fitted distributions of the activity among these groups came from the same distribution. “n” represents the number of independent events in each trophic guild and size. Bottom right panel represents the results at the regional level Figure 4.


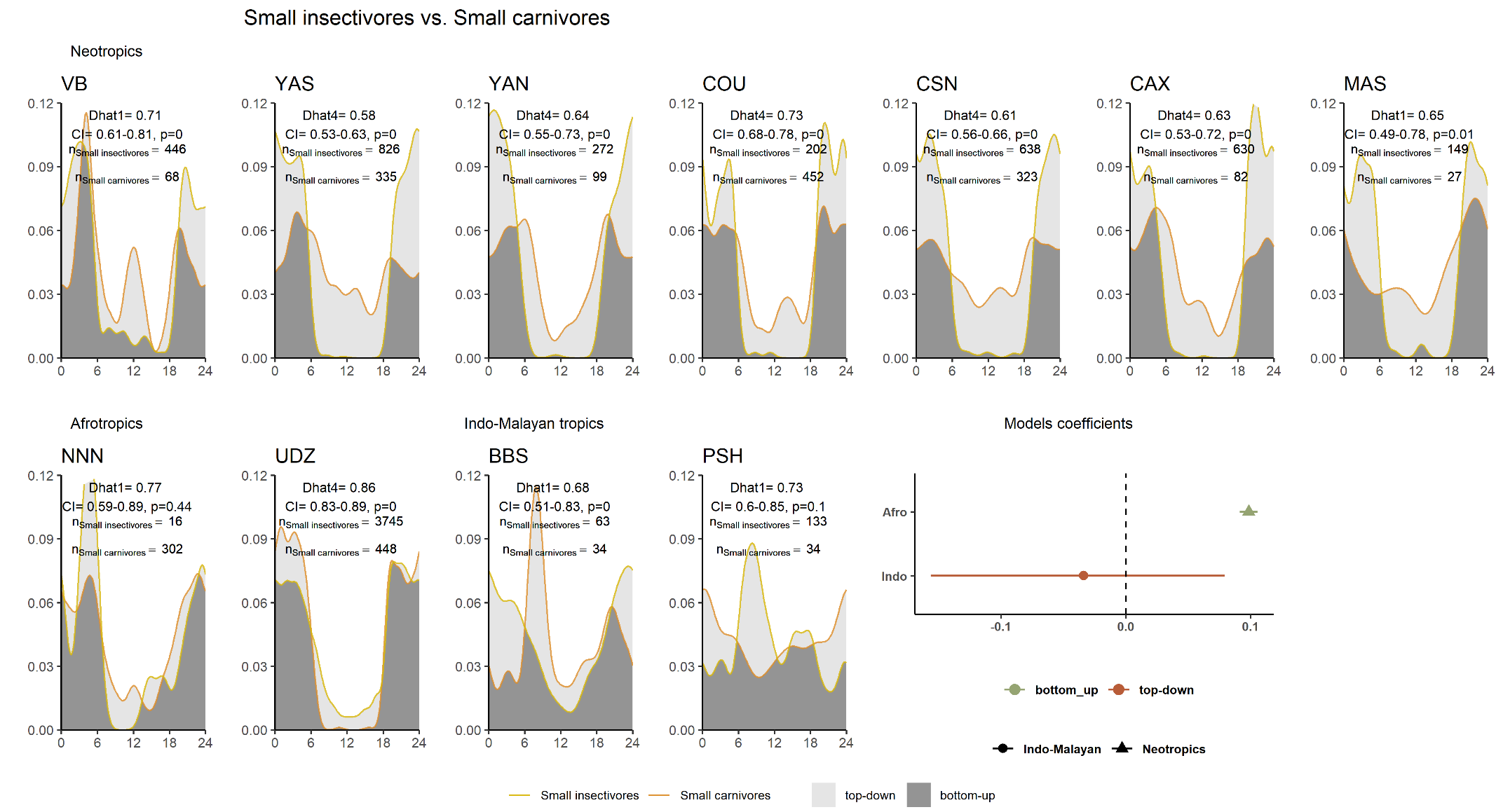


**Figure S11.** Fitted kernel density distribution and coefficient of overlap (Dhat) between the activity of small carnivores vs. small insectivores at the protected area level. Lines: Fitted kernel density distribution, Dhat1: coefficient of overlap type 1 if the sample size was lower than 75, and Dhat4: type 1 if the sample size was higher than 75, CI: 95% confidence intervals, p-value: probability that the fitted distributions of the activity among these groups came from the same distribution. “n” represents the number of independent events in each trophic guild and size. Bottom right panel represents the results at the regional level Figure 4.


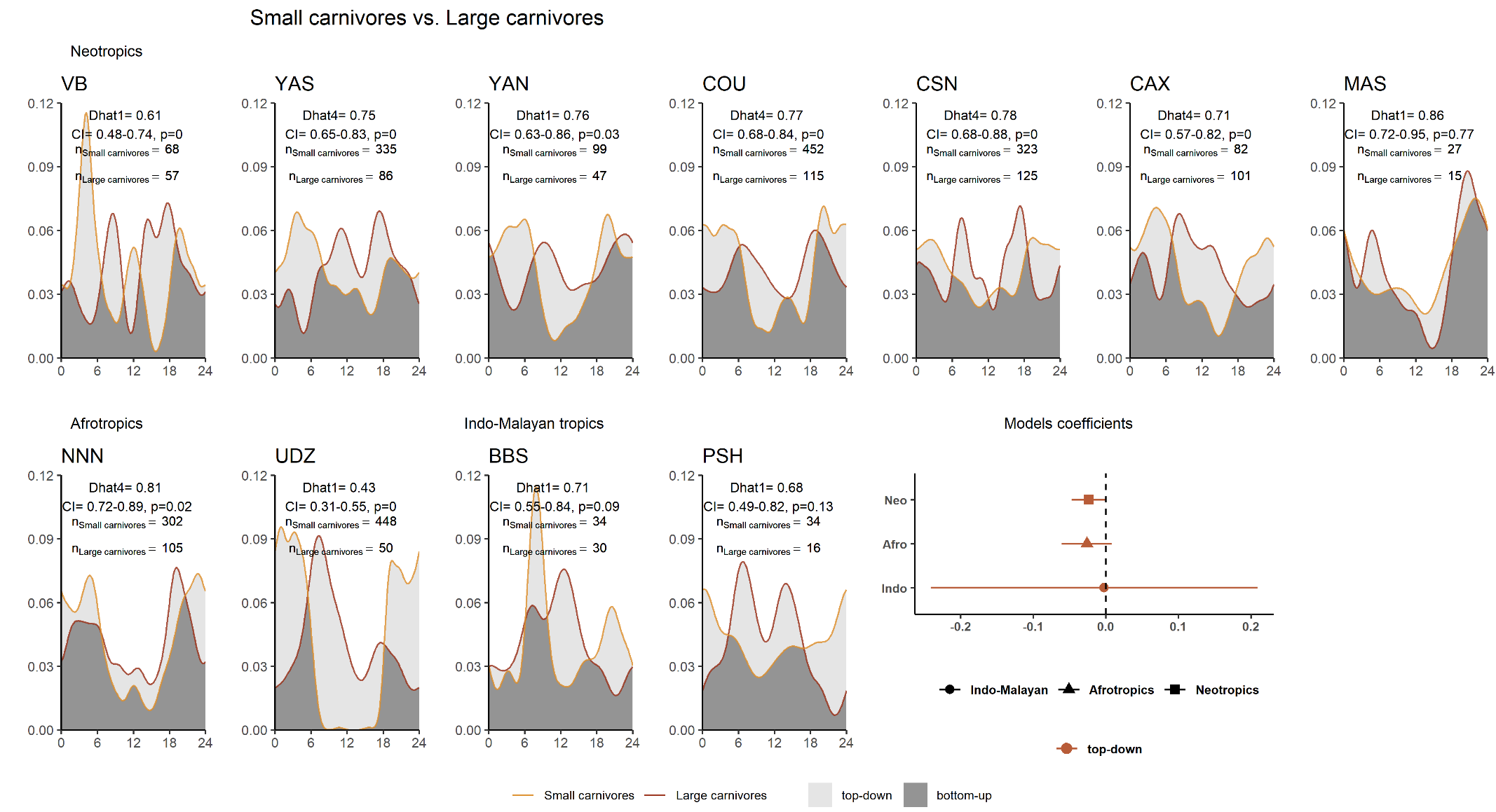


**Figure S12.** Fitted kernel density distribution and coefficient of overlap (Dhat) between the activity of large carnivores vs. small carnivores at the protected area level. Lines: Fitted kernel density distribution, Dhat1: coefficient of overlap type 1 if the sample size was lower than 75, and Dhat4: type 1 if the sample size was higher than 75, CI: 95% confidence intervals, p-value: probability that the fitted distributions of the activity among these groups came from the same distribution. “n” represents the number of independent events in each trophic guild and size. Bottom right panel represents the results at the regional level Figure 4.
