# Supplementary material for: Consistent diel activity patterns of forest mammals among tropical regions
Source: Nat Commun. 2022 Nov 19;13:7102. doi: 10.1038/s41467-022-34825-1 (PMC9675769; doi:10.1038/s41467-022-34825-1)
Supplement: Supplementary file 2 — Reporting Summary [file 41467_2022_34825_MOESM2_ESM.pdf]

nature portfolio

Corresponding author(s): Andrea F. Vallejo-Vargas

Last updated by author(s): Nov 5, 2022

Reporting Summary

Nature Portfolio wishes to improve the reproducibility of the work that we publish. This form provides structure for consistency and transparency in reporting. For further information on Nature Portfolio policies, see our Editorial Policies and the Editorial Policy Checklist.

Statistics

For all statistical analyses, confirm that the following items are present in the figure legend, table legend, main text, or Methods section.

n/a	Confirmed	
☐	☒	The exact sample size (*n*) for each experimental group/condition, given as a discrete number and unit of measurement
☒	☐	A statement on whether measurements were taken from distinct samples or whether the same sample was measured repeatedly
☐	☒	The statistical test(s) used AND whether they are one- or two-sided<br>*Only common tests should be described solely by name; describe more complex techniques in the Methods section.*
☐	☒	A description of all covariates tested
☒	☐	A description of any assumptions or corrections, such as tests of normality and adjustment for multiple comparisons
☐	☒	A full description of the statistical parameters including central tendency (e.g. means) or other basic estimates (e.g. regression coefficient) AND variation (e.g. standard deviation) or associated estimates of uncertainty (e.g. confidence intervals)
☐	☒	For null hypothesis testing, the test statistic (e.g. *F*, *t*, *r*) with confidence intervals, effect sizes, degrees of freedom and *P* value noted<br>*Give P values as exact values whenever suitable.*
☒	☐	For Bayesian analysis, information on the choice of priors and Markov chain Monte Carlo settings
☒	☐	For hierarchical and complex designs, identification of the appropriate level for tests and full reporting of outcomes
☒	☐	Estimates of effect sizes (e.g. Cohen's *d*, Pearson's *r*), indicating how they were calculated

Our web collection on statistics for biologists contains articles on many of the points above.

Software and code

Policy information about availability of computer code

Data collection	Raw data from the Tropical Ecology Assessment and Monitoring (TEAM) is available on Wildlife Insights platform (wildlifeinsights.org). The data from camera-traps was processed with a dynamic software package DeskTEAM (a software package developed by the TEAM network). Species characteristics were extracted form PHYLACINE 1.2, available online at https://doi.org/10.5061/dryad.bp26v20. Species list with reviewed forest strata data are available at https://doi.org/10.5061/dryad.f1vhhmgv0.

Data analysis

Data analysis and graphical presentation were performed using R Statistical and Programming Environment, version R-4.2.1

First, we categorized each independent event in three categories 1) day, 2) twilight, or 3) night. Each event was classified by protected area, location, time, and date to specify the sunrise, sunset, nautical dawn, and dusk using the R library 'maptools' version 1.1-4. Then we fit multinomial models with the package 'mclogit' version 0.9.4.2 three levels of the predictor variable and the independent variables body size and trophic guild. We estimated the probability of diurnal, nocturnal, and twilight activity and the 95% lower and upper confidence intervals for the range of body size by each trophic guild and tropical region with the package 'mpred' version 0.2.4.1 for the model with the best fit.
Second, we fit generalized linear mixed models with the package 'lme4' version 1.1-29. The response variable was the number of pictures by hour for a group of species (e.g., large herbivores) and the predictor variable was the number of pictures hourly for carnivores. We extracted the coefficients and 95% lower and upper confidence intervals for each pairwise comparison.
Density plots to represent the diversity of activity patterns in figure Fig. 1 and supplementary information were performed in R we employed the packages 'overlap' version 0.3.4 and 'activity' version 1.3.2. The study site map was built in ArcGis 10.8.1, and the composed Figure 2 was prepared in Inkscape 1.1.1.
All packages used are described and fully referenced in the methods.
All codes used to generate the presented results are available on in the DataverseNO and is available online at https://doi.org/10.18710/BIGEO7

For manuscripts utilizing custom algorithms or software that are central to the research but not yet described in published literature, software must be made available to editors and reviewers. We strongly encourage code deposition in a community repository (e.g. GitHub). See the Nature Portfolio guidelines for submitting code & software for further information.

Data

Policy information about availability of data

All manuscripts must include a data availability statement. This statement should provide the following information, where applicable:

- Accession codes, unique identifiers, or web links for publicly available datasets
- A description of any restrictions on data availability
- For clinical datasets or third party data, please ensure that the statement adheres to our policy

The data generated in this study have been deposited in the DataverseNO database is available online at https://doi.org/10.18710/BIGEO7. The raw camera-trap data employed in this study can be found in Wildlife Insights (www.wildlifeinsights.org). Species characteristics extracted from PHYLACINE 1.2 are available online at https://doi.org/10.5061/dryad.bp26v20. Species list with reviewed forest strata data are available at https://doi.org/10.5061/dryad.f1vhhmgv0

Field-specific reporting

Please select the one below that is the best fit for your research. If you are not sure, read the appropriate sections before making your selection.

☐ Life sciences ☐ Behavioural & social sciences ☒ Ecological, evolutionary & environmental sciences

For a reference copy of the document with all sections, see nature.com/documents/nr-reporting-summary-flat.pdf

Ecological, evolutionary & environmental sciences study design

All studies must disclose on these points even when the disclosure is negative.

Study description

We employed standardized camera trap data across the tropics to examine diel patterns of mammals in relation to body mass and trophic guild. Also investigate the roles of top-down versus bottom-up processes, and thermoregulatory constraints on diel activity.

Research sample

We employed data of ground dwelling and scansorial species, and >75 g of body mass. We excluded arboreal species and smaller species, because detection of these species is low with camera-traps and could cause bias to our results. For our first analysis on the relationship of diel activity to body mass and trophic guild we employed the data of 411 populations and 16 communities. To examine bottom-up and top-down process on diel activity we included communities where top-predators were detected, thus for this analysis we employed the data of 308 populations and 11 communities. We employed data of ground dwelling and scansorial species, and >75 g of body mass. We excluded arboreal species and smaller species, because detection of these species is low and could cause bias to our results.

Sampling strategy

TEAM camera-trap surveys accomplish the requirements on the sampling length (> 30 days and 2 to 10 years), number of cameras deployed (60-90) to assess among others, species richness, occupancy, and diel activity patterns. To study diel activity patterns of mammals it is recommended to have at least 25 independent records (pictures) of each species to infer their activity pattern. Since our analysis employed broad categories of trophic guilds and sizes to test our hypothesis of bottom-up and top-down processes on the activity of mammals, the camera trap data employed allowed us to investigate these nuances. gate these nuances.

Data collection

TEAM data included in this study was collected from camera-traps across 16 protected areas following a standardized camera-trapping protocol. The data was collected during the dry season between 2008 and 2017. At each protected area, the monitoring ran from two to ten years with the deployment of 60 to 90 cameras annually. Camera-traps were placed at a density of 0.5 - 1 camera/km2 (1 camera every km2 or 1 camera every 2 km2) and remained active for ~30 consecutive days, with a total of 60-89 cameras per protected area.

Timing and spatial scale

Camera-trap data was collected between 2008-05-13 to 2017-11-18 across protected areas during the dry season (i.e., months with < 100 mm average rainfall, in absence of dry season the survey took place during the driest part of the year). The deployment of

	camera-traps during dry season was established to avoid the camera damage during the wet season. In each protected area the survey lasted from 2 to a maximum of 10 years (mean = 6.5 years) with more than 30 days in each period. In each protected area cameras were deployed at a density of one camera per 2 km2 but this density is lower (1 camera per 1 km2) for a few sites <120 km2.
Data exclusions	We excluded data from camera-trap sites with inconsistent date-time stamps.
Reproducibility	The data used here was part of a wildlife camera trap monitoring. Camera-traps were successfully deployed in all sites. No experiments have been done. The reproducibility of this study would depend on large-scale monitor in different protected areas.
Randomization	Our study intent to understand how trophic groups use the different daily categories(day, night, and twilights) so we need this specific groups and this should be not randomize.<br>To assess activity, we categorize every event into day, night, or twilight considering specific hours of sunrise, sunset, nautical dawn, and dusk by location and time of the day.<br>We classified each mammal species into four trophic guilds: carnivore, herbivore, insectivore, or omnivore. Categories were based on diet reported in the PHYLACINE database and we classified as carnivore species feeding on ≥ 80% vertebrates, herbivore species feeding on ≥ 80 % plant materials, insectivore feeding on ≥ 80 % insects, the remaining species were categorized as omnivores (e.g., feeding on vertebrates and fruits).<br>Details can be found in Methods.
Blinding	Not applicable in our study. Camera-traps are triggered by the movement of animals. Thus, blinding for data collection and compilation of these data was not relevant. Experts identified species from the images. The consistency of species identification had several cross checks and quality controls.

Did the study involve field work? ☒ Yes ☐ No

Field work, collection and transport

Field conditions	Fieldwork was carried out in tropical forest across three biogeographic regions. The deployment of camera-traps was during the dry season (i.e., months with < 100 mm average rainfall, in absence of dry season the surveys took place during the driest part of the year). According to the WorldClim data, the average minimum and maximum temperature across all sites was ~19 °C and ~29 °C across all protected areas.
Location	The location of the 16 protected areas studied is shown in the manuscript (Figure 2, Table S1).
Access & import/export	In each protected TEAM managers got the required permits to execute the fieldwork. Currently the data is open access and is available in Wildlife insights https://www.wildlifeinsights.org/
Disturbance	No disturbances were caused in the protected areas.

Reporting for specific materials, systems and methods

We require information from authors about some types of materials, experimental systems and methods used in many studies. Here, indicate whether each material, system or method listed is relevant to your study. If you are not sure if a list item applies to your research, read the appropriate section before selecting a response.

Materials & experimental systems

n/a	Involved in the study
☒	☐ Antibodies
☒	☐ Eukaryotic cell lines
☒	☐ Palaeontology and archaeology
☒	☐ Animals and other organisms
☒	☐ Human research participants
☒	☐ Clinical data
☒	☐ Dual use research of concern

Methods

n/a	Involved in the study
☒	☐ ChIP-seq
☒	☐ Flow cytometry
☒	☐ MRI-based neuroimaging
